# DJ-1 preserves ischemic postconditioning-induced cardioprotection in STZ-induced type 1 diabetic rats: role of PTEN and DJ-1 subcellular translocation

**DOI:** 10.1186/s12964-024-01638-2

**Published:** 2024-05-02

**Authors:** Wei Li, Yan Leng, Yonghong Xiong, Wenyuan Li, Yin Cai, Rui Xue, Rong Chen, Shaoqing Lei, Zhengyuan Xia, Zhongyuan Xia

**Affiliations:** 1 Department of Anesthesiology, Renmin Hospital of Wuhan University, Wuhan, China; 2 State Key Laboratory of Pharmaceutical Biotechnology, Department of Medicine, The University of Hong Kong, Pokfulam, Hong Kong China; 3 Department of Anesthesiology, Renmin Hospital, Hubei University of Medicine, Shiyan, Hubei China; 4 Department of Anesthesiology, Affiliated Hospital of Guangdong Medical University, Guangdong, China; 5 Department of Health Technology and Informatics, The Hong Kong Polytechnic University, Hong Kong SAR, China; 6 Research Center for Chinese Medicine Innovation, The Hong Kong Polytechnic University, Hong Kong SAR, China; 7 Research Institute for Future Food, The Hong Kong Polytechnic University, Hong Kong SAR, China

**Keywords:** Diabetes, DJ-1, Myocardial ischemia/reperfusion injury, Ischemic postconditioning

## Abstract

**Background:**

Ischemic postconditioning (IPostC) has been reported as a promising method for protecting against myocardial ischemia-reperfusion (MI/R) injury. Our previous study found that the infarct-limiting effect of IPostC is abolished in the heart of diabetes whose cardiac expression of DJ-1 (also called PARK7, Parkinsonism associated deglycase) is reduced. However, the role and in particular the underlying mechanism of DJ-1 in the loss of sensitivity to IPostC-induced cardioprotection in diabetic hearts remains unclear.

**Methods:**

Streptozotocin-induced type 1 diabetic rats were subjected to MI/R injury by occluding the left anterior descending artery (LAD) and followed by reperfusion. IPostC was induced by three cycles of 10s of reperfusion and ischemia at the onset of reperfusion. AAV9-CMV-DJ-1, AAV9-CMV-C106S-DJ-1 or AAV9-DJ-1 siRNA were injected via tail vein to either over-express or knock-down DJ-1 three weeks before inducing MI/R.

**Results:**

Diabetic rats subjected to MI/R exhibited larger infarct area, more severe oxidative injury concomitant with significantly reduced cardiac DJ-1 expression and increased PTEN expression as compared to non-diabetic rats. AAV9-mediated cardiac DJ-1 overexpression, but not the cardiac overexpression of DJ-1 mutant C106S, restored IPostC-induced cardioprotection and this effect was accompanied by increased cytoplasmic DJ-1 translocation toward nuclear and mitochondrial, reduced PTEN expression, and increased Nrf-2/HO-1 transcription. Our further study showed that AAV9-mediated targeted DJ-1 gene knockdown aggravated MI/R injury in diabetic hearts, and this exacerbation of MI/R injury was partially reversed by IPostC in the presence of PTEN inhibition or Nrf-2 activation.

**Conclusions:**

These findings suggest that DJ-1 preserves the cardioprotective effect of IPostC against MI/R injury in diabetic rats through nuclear and mitochondrial DJ-1 translocation and that inhibition of cardiac PTEN and activation of Nrf-2/HO-1 may represent the major downstream mechanisms whereby DJ-1 preserves the cardioprotective effect of IPostC in diabetes.

**Graphical abstract:**

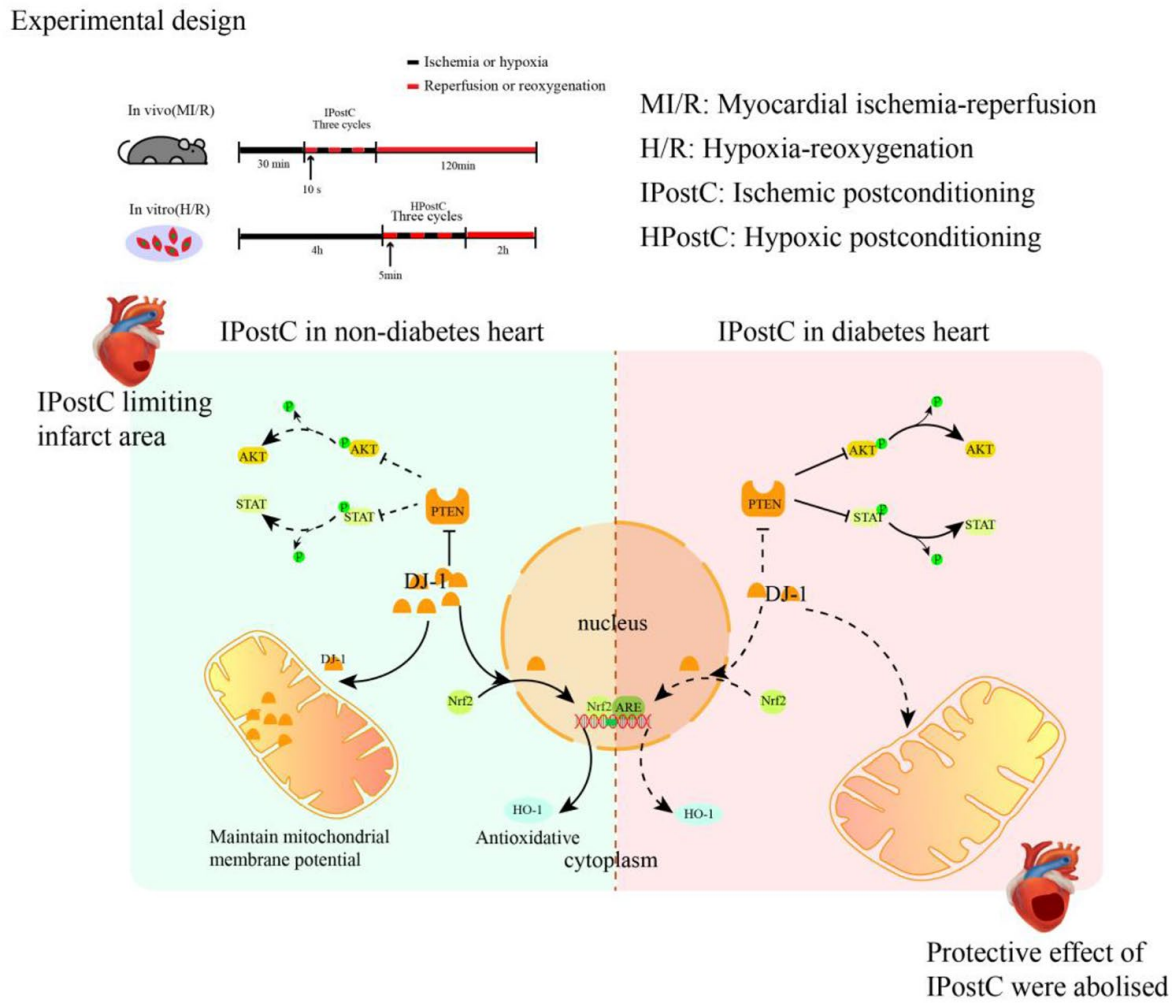

**Supplementary Information:**

The online version contains supplementary material available at 10.1186/s12964-024-01638-2.

## Background

Cardiovascular complications occur more often in diabetic patients and patients with diabetes are more vulnerable to cardiac ischemic insult, which is a leading cause of death in patients with diabetes [1]. Ischemic postconditioning (IPostC), a series of brief ischemia and reperfusion cycles applied immediately upon reperfusion at the site of the ischemic organ, has been shown to be an optimal therapy for myocardial ischemia-reperfusion (MI/R) injury in non-diabetic subjects [2]. IPostC has been shown to protect the myocardium from ischemia-reperfusion (I/R) injury by reducing oxidative stress in the hearts of nondiabetic animals but not in those of diabetic animal models due to insufficient activation of the antioxidative stress pathway. However, evidence from our previous study [3, 4] and others [5] suggests that the protective effects of IPostC are significantly diminished or even abolished in the diabetic myocardium, but the underlying mechanisms remain unclear.

The protein DJ-1 (also called PARK7), a redox-sensitive molecular chaperone that protects cells from oxidative stress through its nuclear and mitochondrial DJ-1 translocation, is ubiquitously expressed in tissues and is mainly localized in the cytoplasm, nucleus and mitochondrial [6]. The cysteine residue at position 106 (C106) of DJ-1 is highly sensitive to oxidative stress and crucial for the activation of DJ-1. Nuclear DJ-1 binds to many proteins, including various transcription factors, and acts as a coactivator or corepressor to regulate its target genes without direct binding to DNA, thereby affecting various cellular functions. Our previous study [7] showed that DJ-1 was reduced in diabetic heart that was coincident with diabetic heart increased vulnerability to MI/R injury. The paradox is that DJ-1 overexpression in the diabetic heart provides very little protection against I/R injury. While DJ-1 overexpression combined with IPostC markedly reduced MI/R-induced damage [8]. However, the underlying mechanism by which DJ-1 activation potentiates or restores IPostC protective effects in the diabetic myocardium remains unclear.

Phosphatase and tensin homolog deleted on chromosome ten (PTEN) was originally discovered as a novel tumor suppressor gene and was subsequently found to be a major negative regulator of the pro-survival PI3K/Akt signaling pathway, affecting cell proliferation, growth apoptosis and immunity [9]. We recently showed that PTEN increased in diabetic hearts that was associated with increased MI/R injury [10] and diminished diabetic heart responsiveness to IPostC [11]. Further, inhibition of PTEN could restore diabetic heart responsiveness to IPostC-induced cardioprotection that was associated with increased PI3K/Akt activation [11], an effect that was similar to that seen in diabetic heart that DJ-1 overexpression restores IPostC as aforementioned. However, whether or not and how DJ-1 and PTEN may interact to affect IPostC effectiveness in diabetes is unknown.

DJ-1 has been found to be associated with PTEN in mesangial cell hypertrophy, and the interaction between DJ-1 and PTEN is significantly increased in response to high-glucose (HG) conditions [12]. Furthermore, we previously showed [13, 14] that the signaling pathway associated with Nrf-2, a key nuclear transcription factor involved in endogenous protection against cellular oxidative stress processes [15], is impaired in the diabetic heart that is attributable to the loss of responsiveness to postconditioning cardioprotection [13]. Studies conducted in animal models of diabetic nephropathy and of Parkinson’s disease have shown that expression of DJ-1 may effectively combat oxidative stress by inhibiting PTEN and stabilizing Nrf-2 [16, 17]. We, thus, hypothesized that DJ-1 reduction in the diabetic heart is responsible for the reduced diabetic heart sensitivity to IPostC and it does so via inhibiting PTEN and the subsequent activation of Nrf-2 signaling.

## Methods

### Experimental animals

Healthy SPF-grade male adult Sprague-Dawley rats weighing 200 ± 10 g (6–8 weeks of age) were supplied by Beijing HFK Bioscience Co. Ltd. All the rats were housed in an environment with a controlled temperature of 24℃, relative humidity of 50 ± 10%, and a fixed light/dark schedule (12 h (h) light/12 h dark) and were allowed free access to food and water. All the rats were given free access to standard care in accordance with the principles of Animal Care of Wuhan University, and the experimental protocols were approved by the Wuhan University Bioethics Committee for the Use of Live Animals in Teaching and Research.

### Establishment of diabetes model

After 3 days of acclimatization, all rats were fasted 12 h before diabetes induction. The Type 1 diabetes model was induced by a single intraperitoneal injection of 60 mg/kg STZ (streptozotocin) (Sigma-Aldrich, USA) dissolved in citrate buffer (Dissolve 2.10 g of citric acid and 2.94 g of sodium citrate in 100 ml of double-distilled water to obtain citric acid solution A and sodium citrate solution B. When used, prepare the citric acid-sodium citrate buffer (0.1 M) according to the ratio of A: B = 1:1.32, with a pH of 4.5.). As a control, normal rats were administered the same amount of citrate buffer alone. After 72 h (with 6 h fasting), rats with a fasting blood glucose level ≥ 16.7 mmol/L was considered diabetic.

### Preparation of myocardial ischemia reperfusion model

In vivo, the MI/R injury model was achieved by occluding the LAD (left anterior descending) artery for 30 min (min), followed by 120 min of reperfusion. IPostC was induced by three cycles of 10 s (s) of reperfusion and ischemia at the onset of reperfusion. Sham-operated groups underwent the same surgical procedures without LAD ligation. The criteria of successful establishment of ischemia were as follows: the apical and anterior wall of the left ventricle became white, the ECG showed a widened QRS complex, the ST segment was elevated, height tip of the T wave was heightened, and the ventricular wall motion decreased. The criteria of successful reperfusion were as follows: the apex and anterior wall of the left ventricle turned red and ventricular wall motion recovered, and the ECG showed a normal ST. Blood and tissue samples were collected for further analyses at the end of reperfusion.

### Experimental protocols

After 8 weeks of diabetes induction, both DM (diabetic) and age-matched N (non-diabetic) rats were randomly divided into one of the following six groups (*n* = 12 in each group): (i) N + S (sham), (ii) N + I/R, (iii) N + IPostC, (iv) DM + S, (v) DM + I/R and (vi) DM + IPostC Groups in the initial set of study. Furthermore, in order to evaluate the endogenous protective mechanism of DJ-1 regulation in diabetic myocardium, a second set of experiments were performed on the groups treated as follows: (i) DM + I/R, (ii) DM + IPostC, (iii) DM + I/R + AAV9 (adeno-associated virus 9)-CMV-DJ-1, (iv) DM + IPostC + AAV9-CMV-DJ-1, (v) DM + IPostC + AAV9-CMV-C106S DJ-1, (vi) DM + I/R + AAV9–DJ-1 siRNA, (vii) DM + IPostC + AAV9-DJ-1 siRNA, (viii) DM + IPostC + AAV9-DJ-1 siRNA + BpV (Bisperoxovanadium) and (ix) DM + IPostC + AAV9-DJ-1 siRNA + Otz(Oltipraz). The PTEN inhibitor BpV (1.0 mg/kg dissolved in 1%DMSO) (Alexis/Enzo Life Science) and vehicle (0.9%NaCl dissolved in 1% DMSO), which served as a control, were respectively administered via femoral vein injections 1 h before ischemia (LDA occlusion). And, the Nrf2 activator Otz (10 mg/kg dissolved in 1% DMSO) and vehicle (0.9%NaCl dissolved in 1% DMSO), were also administered via femoral vein injections 1 h before ischemia (LDA occlusion).

### AAV infection

AAV9 vectors, which carry a CMV promoter for high-level gene expression of DJ-1 or DJ-1 siRNA for reducing the gene expression of DJ-1, were produced by Hanbio Biotechnology Co. AAV9-CMV、AAV9-CMV-DJ-1、AAV9-CMV-C106S DJ-1、AAV9-siRNA or AAV9-DJ-1 siRNA was administered via tail vein injection at a dose of 2 × 10^12^ vg/kg three weeks before myocardial I/R induction (that is five weeks after diabetes induction). Western Blot and qt-RNA are used to detect transfection and expression efficiency.

### Cardiac function assessment

To evaluate left ventricle myocardial function, a saline-filled catheter was inserted into the left ventricle via an incision on the right common carotid artery, and then connected to a pressure transducer (Yixinda, China). The LVSP (left ventricular systolic pressure), left ventricular + dp/dt (maximum rate of increase of left ventricular developed pressure), −dp/dt (maximum rate of decrease of left ventricular developed pressure) and HR (heart rate) were continuously monitored by electrophysiology (MH150, Biopac) at 10 min before inducing ischemia (baseline) and at 120 min after reperfusion (I/R insult or IPostC procedure). The data were derived by AcqKnowledge 4.0 software.

### Determination of myocardial infarct size

At the end of the reperfusion, the sizes of the infarct area (IA) and area at risk (AAR) of the experimental rats was measured using 3% Evans Blue dye (Sigma-Aldrich) and 1% 2,3,5-triphenyltetrazolium chloride (pH 7.4) (Sigma-Aldrich) staining. The infarct size was determined by using an image analysis system (Image-Pro Plus 3.0; Media Cybernetics, MA). Two researchers independently scored the heart slides to ensure reliability of the results. The blue area was normal myocardium, red indicated ischemic myocardium, pale denoted myocardial infarction, and the percentage of IA versus AAR (IA/AAR ×100%) was calculated.

### H9c2 cells culture

Rat cardiomyocyte-derived cell line H9c2 was obtained from the A.T.C.C. (Manassas, VA, U.S.A.). Cells were maintained in DMEM (Dulbecco’s modified Eagle’s medium) (Gibco Laboratories) containing 10% (v/v) FBS (Gibco Laboratories) and 100 µg/ml penicillin/streptomycin (Gibco Laboratories) under an atmosphere of 10% CO_2_ and 90% air at 37℃. The cells were randomly divided into the following groups: (i) LG (low-glucose(5.5mM) medium), (ii) LG + H/R (hypoxia-reoxygenation), (iii) LG + HPostC (hypoxia post-conditioning), (iv) HG (high glucose) (30 mM) medium, (v) HG + H/R, (vi) HG + HPostC. To determine whether DJ-1 overexpression preserves HPO-induced protection and how it works, further experiments were performed under the following conditions: (i) HG + H/R, (ii) HG + HPostC, (iii) HG + H/R + pcDNA3.1-DJ-1-Flag (GenePharma), (iv) HG + HPostC + pcDNA3.1-DJ-1-Flag, (v) HG + HPostC + pcDNA3.1- C106S DJ-1-Flag(GenePharma), (vi) HG + H/R + DJ-1siRNA, (vii) HG + HPostC + DJ-1siRNA, (viii) HG + HPostC + DJ-1siRNA + BpV, and (ix) HG + HPostC + DJ-1siRNA + Otz. H9c2 cells were seeded in the appropriate culture plates, pcDNA3.1-DJ-1-Flag plasmids、pcDNA3.1-C106S DJ-1-Flag plasmids or DJ-1siRNA was transfected into cells for DJ-1/C106S D-1 gene overexpression or knockdown, and pcDNA3.1 plasmids、Scramble siRNA or DMSO (less than 0.1%, inhibitor solvent) was used as control according to the manufacturer’s instructions respectively. When cells reached 60-70% confluence, they were placed in serum-free defined medium containing 0.1% BSA overnight, and then exposed to HG for 48 h. BpV(15µM) or Otz (25mM) was administered 1 h before hypoxia induction respectively, then H/R was established by 4 h of hypoxia (10% CO_2_、89% N_2_ and 1% O_2_) followed by 2 h of reoxygenation (10% CO_2_ and 90% air). HPostC was achieved by three cycles of 5 min of reoxygenation and hypoxia after 4 h of hypoxia, then followed by 2 h of reoxygenation. Mannitol (Sigma-Aldrich) (24.5mM) was added in the control group to exclude hyperosmolar effects. The cells and culture medium were collected for subsequent experiments. Each experiment was performed six times independently in triplicate.

### Cell viability assay

Cell viability was determined in 96-well plates using CCK-8(cell counting kit-8)Assay Kit (Dojindo, Kumamoto, Japan) according to manufacturer’s instructions. The absorbance was measured at 450 nm by using a microplate reader. The mean optical density (OD) of 6 wells in each group was used to calculate the percentage of viable cells with the following formula: cell viability = treatment group OD/control group OD × 100%.

### Measurement of LDH (Lactate dehydrogenase) and CK-MB (Creatine Kinase-MB) activity

For the in vivo study, arterial blood samples were collected at the end of reperfusion and centrifuged to collect the serum (2000 rpm, 10 min). For the in vitro study, the supernatant of the cell culture was collected. The release of lactate dehydrogenase (LDH) (Jiancheng, Nanjing, China) and creatine kinase-MB (CK-MB) (Beyotime Biotechnology, China) were measured using assay kits according to the manufacturer’s instructions as we reported [18].

### Measurement of O_2_^−^, superoxide dismutase (SOD), lipid peroxidation and 15-F2t-isoprostane levels

To examine O_2_^−^ production in cardiac tissues or H9c2 cells, the lucigenin chemiluminescence method was utilized. Briefly, after H/R, the supernatant samples were collected and loaded with 5 µM dark-adapted lucigenin; subsequently, light emission was detected by using a luminometer (GloMax, Promega) for 30 min at room temperature. Light emission was recorded every 5 min. The results are expressed as the mean light units (MLU)/min/100 µg protein. Superoxide Dismutase (SOD) and lipid peroxidation (LPO) concentrations were analyzed spectrophotometrically according to the manufacturer’s instructions (Nanjing Jiancheng Bioengineering Institute, Nanjing, China). Furthermore, 15-F2t-isoprostane was used as a special index of oxidative stress-induced lipid peroxidation in MI/R injury, which were measured using an ELISA kit (Cayman Chemical) as we reported [19].

### Mitochondrial permeability transition pore (mPTP) in cardiomyocytes

To examine the mPTP opening, H9c2 cardiomyocytes were stained with the mPTP fluorescence (Genmed Scientifics Inc, MA, USA) and assessed as described [20]. H9c2 cells were loaded with 0.25 mM calcein-acetoxymethylester (calcein-AM) and 8 mM cobalt chloride at 37℃ for 20 min. Fluorescence signal was observed by fluorescence microscope (Olympus, Bx 50-FLA) at 488 nm excitation and 525 nm emission. The results were represented as relative fluorescence intensity. The average fluorescence intensity was analyzed by Image-Pro advanced software.

### Mitochondrial membrane potential(Δψm) assessment

The cells were incubated with 5 mg/ml JC-1 dye (Beyotime Bio) for 30 min in darkness. JC-1 accumulates and aggregates in the mitochondria and selectively exhibits a red emission profile, which appears in the matrix of actively respiring mitochondria. Depolarization of the mitochondrial membrane decreases membrane potential, resulting in emission of green fluorescent JC-1 monomer. Images were acquired using an Olympus microscope (Olympus Corporation), and the green/red average fluorescence intensity was calculated using Image-Pro Plus software.

### ROS measurement by DHE staining

ROS detection was based on the oxidation of the superoxide indicator DHE (dihydroethidium) to ethidium. Briefly, cardiomyocytes were incubated with 2 ml of 10 µmol/l DHE (Molecular Probes/Invitrogen) at 37 ℃ for 30 min in darkness, and then washed twice using cold PBS. DHE exhibits blue fluorescence in the cytosol until oxidized, where it intercalates within the cell’s DNA, staining its nucleus a bright fluorescent red. Images were captured using a fluorescence microscope (Olympus IX51). The average fluorescence intensity was analyzed using an Image Pro advanced software.

### Mitochondrial ROS staining assay

Mitochondrial ROS production was detected by Mito Sox Red (Invitrogen, Carlsbad, CA) staining as described previously [21]. After the stimulation with isoproterenol (100 µM, 48 h) in the presence or absence of canstatin (10–250 ng/ml, 30 min pretreatment), cells were washed and treated with 5 µM Mito Sox Red solution in Hank’s Balanced Salt Solution for 10 min at 37 °C. Images were obtained by a cooled CCD camera (MicroPublisher 5.0 RTV)-equipped fluorescence microscope (BX-51, OLYMPUS, Tokyo, Japan). Fluorescence intensity of mitochondrial ROS was measured by an Image J software (National Institutes of Health, Bethesda, MD).

### Determination of apoptosis

For the in vivo study, TUNEL (terminal deoxynucleotidyl transferase-mediated dUTP nick-end labelling) was used to assess cardiac tissue apoptosis with an in situ cell death detection kit (Roche Diagnostics). TUNEL-positive cells displayed brown staining within the nucleus of apoptotic cells, and the apoptotic index was calculated as a percentage of apoptotic nuclei to total nuclei according to the manufacturer’s protocol (Roche, Indianapolis, USA). Ten fields for each sample were randomly chosen to determine the apoptosis rate. For the in vitro study, H9c2 cells were collected and resuspended in binding buffer, then incubated with 5 µl of FITC (fluorescein is thiocyanate)-conjugated annexin V and 10 µl of 1 mg/ml PI (propidium iodide) for 10 min in darkness. Cellular fluorescence was subsequently measured by using a FACS Calibur flow cytometer (BD Biosciences) and the data obtained from the cell population were analyzed with Cell Quest Pro software (BD Biosciences). Furthermore, caspase-3 is a pivotal mediator of apoptosis; myocardial caspase-3 expression was assessed specifically by Western botting assay as stated below.

### Myocardial ATP assay

After reperfusion, hearts were removed rapidly and 100 mg ventricle tissue was dissected and homogenized in 1 ml ice-cold PBS buffer containing protease and phosphatase inhibitor cocktails. Myocardial ATP levels were measured using an ATP bioluminescence assay kit (Beyotime, China) in accordance with manufacturer’s instructions. The emitted light was linearly related to the ATP concentration and measured using a microplate luminometer.

### PTEN activity

The PTEN Activity ELISA is designed to quantify the phosphatase activity of PTEN by detection of the product, PIP2, in a competitive ELISA format, eliminating the need for radioactivity, organic solvents, and thin layer chromatography PTEN activity was detected according to the assay kit instructions (K-4700-1kit, Echelon, USA).

### Western blot analysis

The levels of proteins extracted from left ventricular tissue samples or cultured H9c2 cells were measured with Western blot assay as described previously (REF). Briefly, nuclear, cytoplasmic and mitochondrial extracts of left ventricular tissue and H9c2 cells were prepared using nuclear and cytoplasmic extraction reagent (Beyotime, China) as recommended by the manufacturer. Primary antibodies against DJ-1, PTEN, Akt, p-Akt (Ser473), Nrf-2, HO-1, STAT3, p-STAT3 (Tyr705), cleaved-Caspase-3, Bax, Bcl-2, Lamin b1, COX IV or GAPDH (glyceraldehyde-3-phosphate dehydrogenase) (1:1000 dilution, Cell Signaling Technology) were used. Lamin b1, COX IV or GAPDH served as a loading control to ensure equal loading. Signals were detected using a fluorescence imaging scanner (Odyssey, Germany) and optical densities were quantified using the Odyssey Image Analysis Software. The immunoblot assays were replicated three times for each protein, with left ventricular samples or cultured H9c2 cells (*n* = 6) per group.

### Statistical analysis

The experimental rats and H9c2 cell culture dishes were randomly assigned to treatment or control groups. All data are expressed as the mean ± SD. Comparisons between multiple groups were made by one-way ANOVA followed by the Tukey test. Statistical analysis was performed using GraphPad Prism 6.0 for Windows (GraphPad Software, USA). P values less than 0.05 were considered statistically significant.

## Results

### Infarct-limiting effect of IPostC was abolished in the heart of diabetes concomitant with reduced DJ-1expression

As shown in Tables 1 and 8 weeks after STZ injection, rats exhibited characteristic symptoms of diabetes, including hyperglycemia, polydipsia and polyphagia manifested as increased levels of blood glucose, water intake and food consumption as compared with those in the age-matched non-diabetic rats.

**Table 1:** General characteristics of each group after 8 weeks

Parameters /Group	Non-diabetes	Diabetes	Diabetes + AAV9-CMV-DJ-1	Diabetes + AAV9-CMV-C106S-DJ-1
Blood glucose(mM)	5.87 ± 2.68	28.52 ± 4.95^*^	25.52 ± 2.27^*^	27.39 ± 3.35^*^
Body weight (g)	459.5 ± 35.42	254.8 ± 22.65^*^	284.8 ± 24.42^*^	272.35 ± 25.71^*^
Water intake (ml/kg/day)	125.0 ± 14.6	855.0 ± 93.4^*^	739.0 ± 85.4^*^	837.0 ± 92.6^*^
Food consumption (g/kg/day)	75. 4 ± 9.7	203.9 ± 24.1^*^	181.3 ± 27.6^*^	188.4 ± 22.3^*^

AAV9-CMV-DJ-1 or AAV9-CMV-C106S-DJ-1was administered via tail vein injection at a dose of 2 × 10^12^ vg/kg 3 weeks before myocardial IR induction. Results are expressed as mean ± S.D. *p < 0.05 versus Non-diabetes group, *n* = 12

**Fig. 1:**
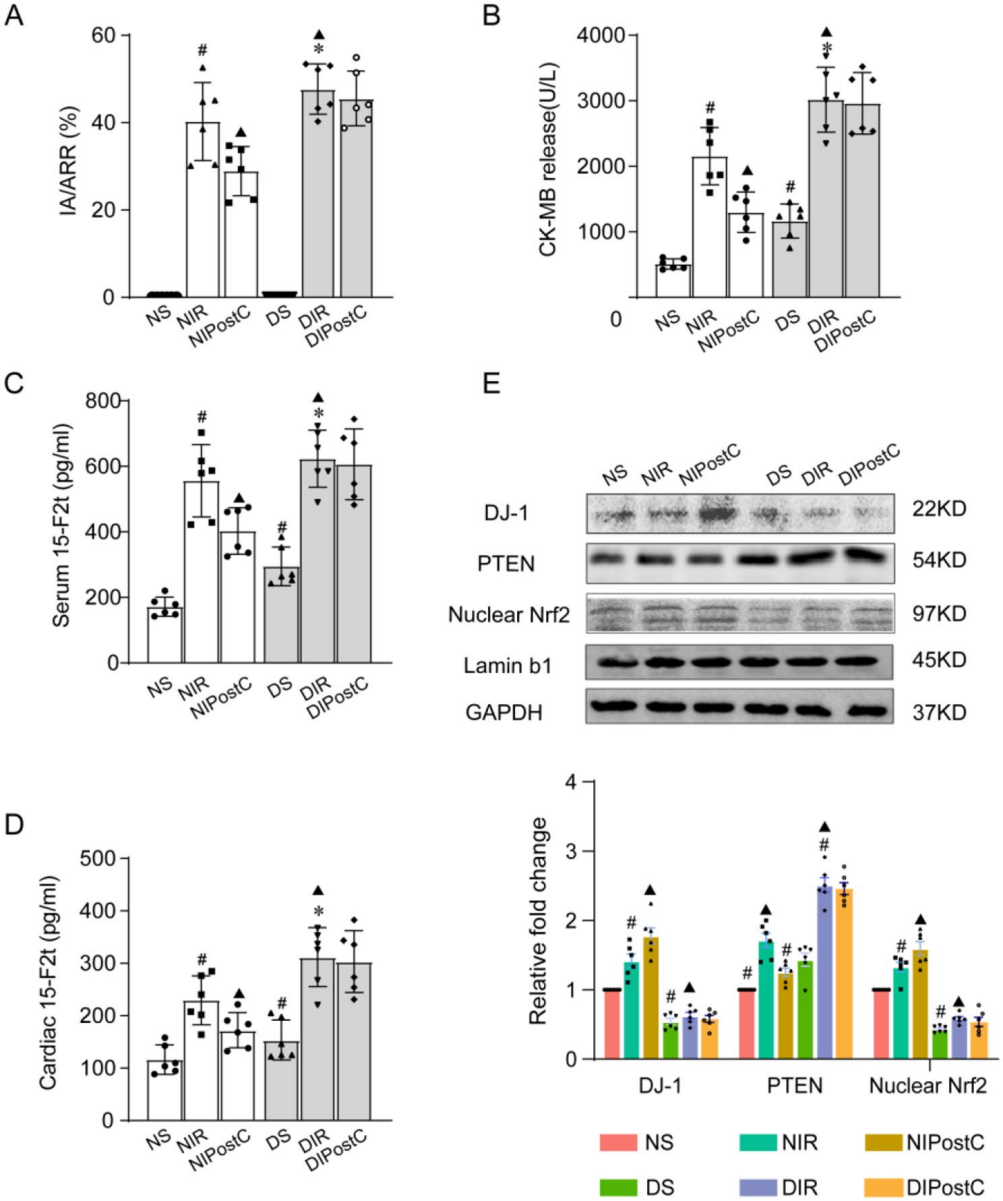
Infarct-limiting effect of IPostC is abolished in the heart of diabetes concomitant cardiac expression of DJ-1 is reduced (**A**) The myocardial infarction area was detected by TTC. (**B**) The CK-MB in serum was detected. (**C**–**D**) The serum and cardiac levels of 15-F2t-isoprostane was detected. (**E**) Representative Western blot images of DJ-1, PTEN and nuclear Nrf2 expressions in the myocardium of the 6 groups of rats. Values are expressed as mean ± SD (*n* = 6 per group). ^#^*P* < 0.05 vs. NS group, ${^{\blacktriangle}}\;P$ < 0.05 vs. NIR group, ^*^P < 0.05 vs. DS group

IPostC significantly decreased the infarct size in non-diabetic rats but failed to protect diabetic rats from myocardial ischemic injury. In addition, after MI/R injury, the infarct size **(**Fig. 1A**)**, CK-MB **(**Fig. 1B**)**, LDH (Supplement 1A) and serum (Fig. 1C**)** and cardiac 15-F2t-isoprostane levels **(**Fig. 1D**)** in the diabetic hearts were significantly higher than that in non-diabetic rats. Furthermore, as shown in Table 2, after MI/R, HR, LVSP, +dp/dt max and -dp/dt max levels in the diabetic and non-diabetic rats were both significantly lower than the corresponding baseline levels. Among them, HR, LVSP, +dp/dt and -dp/dt changes were reversed by IPostC in age-matched no-diabetic control rats, but not in diabetic rats.

**Table 2:** Haemodynamic parameters reflecting left ventricular function in diabetic rats after 2 h of reperfusion

	Baseline	Reperfusion	Reperfusion with IPO
**HR (min** ^− **1**^ **)**			
Non-DM	391 ± 32	259 ± 37^##^	322 ± 28**
DM	289 ± 25	182 ± 31^##^	193 ± 34
DM + DJ-1^+^	308 ± 32	212 ± 27^##^	277 ± 24^ΔΔ^**
DM + DJ-1^+^mutant	305 ± 29	197 ± 29^##^	217 ± 27
DM + DJ-1^−^	258 ± 27	164 ± 36^##^	162 ± 38
DM + DJ-1^−^+Bpv	249 ± 32	175 ± 23^##^	${{203\pm 27 ^{\blacktriangle}}{^{\blacktriangle} }{^{\ast}}}$
DM + DJ-1^−^+Otz	241 ± 35	169 ± 26^##^	$237 \pm 31 {^{\blacktriangle \blacktriangle \ast \ast}}$
**LVSP (mmHg)**			
Non-DM	135 ± 17	92 ± 13^##^	114 ± 14**
DM	97 ± 11	49 ± 8^##^	52 ± 7
DM + DJ-1^+^	113 ± 12	59 ± 8^##^	83 ± 10^ΔΔ^**
DM + DJ-1^+^mutant	108 ± 8	53 ± 10^##^	56 ± 11
DM + DJ-1^−^	86 ± 12	44 ± 6^##^	45 ± 8
DM + DJ-1^−^+Bpv	87 ± 11	53 ± 11^##^	$72 \pm 9 {^{\blacktriangle \blacktriangle \ast \ast}}$
DM + DJ-1^−^+Otz	85 ± 13	49 ± 9^##^	$62 \pm 11 {^{\blacktriangle}}{^{\ast}}$
**+dp/dt (mmHg/s)**			
Non-DM	6327 ± 954	4725 ± 607^##^	5620 ± 485**
DM	4652 ± 571	2389 ± 352^##^	2362 ± 289
DM + DJ-1^+^	4810 ± 465	2507 ± 382^##^	4013 ± 390^ΔΔ^**
DM + DJ-1^+^mutant	4730 ± 570	2422 ± 421^##^	2498 ± 307
DM + DJ-1^−^	4355 ± 502	2824 ± 329^##^	2705 ± 277
DM + DJ-1^−^+Bpv	4270 ± 478	3052 ± 341^##^	$3672 \pm 375 {^{\blacktriangle \blacktriangle \ast \ast}}$
DM + DJ-1^−^+Otz	4378 ± 492	2980 ± 294^##^	$3784 \pm 386 {^{\blacktriangle \blacktriangle \ast \ast}}$
**-dp/dt (mmHg/s)**			
Non-DM	5283 ± 804	3327 ± 446^##^	4256 ± 608**
DM	3694 ± 485	2128 ± 343^##^	2205 ± 304
DM + DJ-1^+^	3806 ± 467	2375 ± 365^##^	3161 ± 347^ΔΔ^**
DM + DJ-1^+^mutant	3775 ± 514	2275 ± 409^##^	2358 ± 425
DM + DJ-1^−^	3428 ± 429	1957 ± 317^##^	2033 ± 315
DM + DJ-1^−^+Bpv	3443 ± 412	2158 ± 359^##^	$2611 \pm 417 {^{\blacktriangle}}^{\ast}$
DM + DJ-1^−^+Otz	3380 ± 439	2107 ± 354^##^	$2764 \pm 385 {^{\blacktriangle \blacktriangle \ast \ast}}$

Results are expressed as mean ± SD.^##^*p* < 0.01 vs. Baseline,^#^*p* < 0.05 vs. Baseline, **p < 0.01 vs. Ischemia/reperfusion group, *p < 0.05 vs. Ischemia/reperfusion group,^ΔΔ^*p* < 0.01 vs. DM + IPO group,^Δ^*p* < 0.05 vs. DM + $\text{IPO group},\;^{\blacktriangle \blacktriangle}$*p* < 0.01 vs. DM + DJ-1^−^+$\text{IPO group},\;^{\blacktriangle}$*p* < 0.05 vs. DM + DJ-1^−^+IPO group

As shown in Fig. 1E, DJ-1 expression was significantly lower in diabetic than in non-diabetes. Notably, IPostC significantly upregulated DJ-1 expression in NIPostC group but not in DIPostC group. The cardiac protein levels of p-Akt (Supplement 1B) and p-STAT3 (Supplement 1C) and nuclear Nrf-2**(**Fig. 1E**)** and HO-1 (Supplement 1D) were significantly decreased in diabetic rats compared to non-diabetic rats, while PTEN **(**Fig. 1E**)** was significantly elevated. Furthermore, MI/R insult increased nuclear Nrf-2 **(**Fig. 1E**)** and HO-1 expression and induced Akt and STAT3 phosphorylation in non-diabetic rats (Supplement 1B-C), and these effects were further enhanced by IPostC; however, these alterations were not observed in diabetic rats.

### Subcellular location of DJ-1 is crucial for the protective effects of IPostC in diabetic heart

To determine whether diabetes induced DJ-1 inhibition impairs IPostC-induced cardioprotection in diabetic rats, we overexpressed DJ-1 in myocardial tissue of diabetic rats by AAV9-CMV-DJ-1 injections. We confirmed that 3 weeks after AAV9-CMV-DJ-1 infection, the protein expression level of DJ-1 in the treated group was nearly 3.2 times higher than that in the control group (Supplement 2B).

We next determined the effect of DJ-1 and its mutant (C106S) overexpression on post-ischemic cardiac dysfunction in diabetic hearts. As shown in Table 2, DJ-1 overexpression alone slightly increased HR, LVSP, +dp/dt or -dp/dt but the differences did not reach statistical significance. However, in the presence of DJ-1 overexpression, IPostC significantly improved HR, LVSP, +dp/dt and -dp/dt in diabetic rats (all *p* < 0.05 vs. diabetic untreated), which is indicative that DJ-1 overexpression restored IPostC cardioprotection in diabetes.

**Fig. 2:**
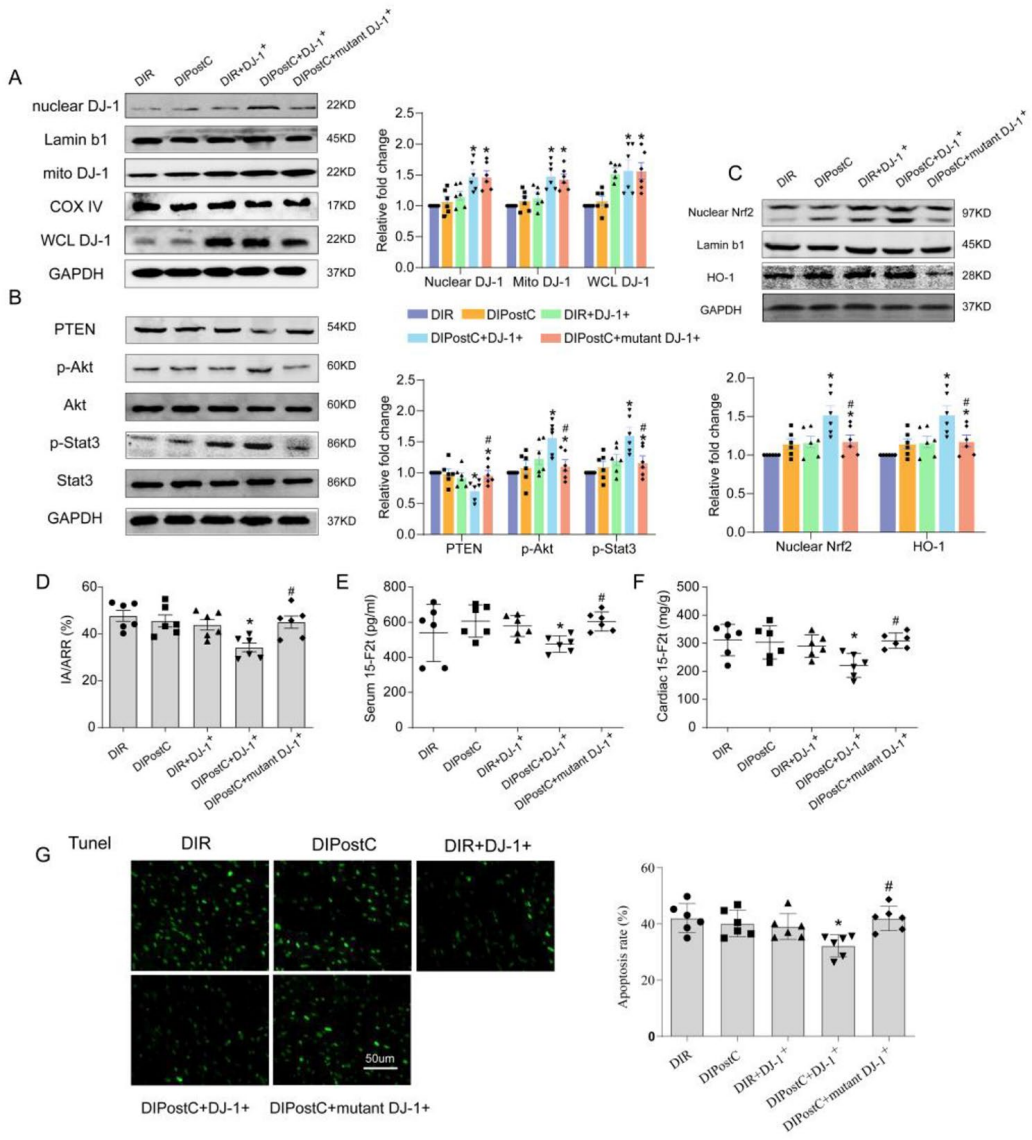
Subcellular location of DJ-1 is crucial for the protective effects of IPostC in diabetic heart (**A**) Representative Western blot images of mitochondrial, nuclear and whole cell (WCL) DJ-1 expressions in the myocardium of the 5 groups of rats. (**B**) Representative Western blot images of PTEN, p-AKT and p-STAT3 expressions in the myocardium of the 5 groups of rats. (**C**) Representative Western blot images of nuclear Nrf2 and HO-1 expressions in the myocardium of the 5 groups of rats. (**D**) The myocardial infarction area was detected by TTC. (**E**-**F**) The serum and cardiac levels of 15-F2t-isoprostane was detected. (**G**) The tunnel staining for cell apoptosis rates were detected in different rat groups. Values are expressed as mean ± SD (*n* = 6 per group). *P < 0.05 vs. DIR group, ^**#**^*P* < 0.05 vs. DIPostC + DJ-1^+^ group

To further investigate whether or not DJ-1 relies on its C106S residues to restore IPostC protection in diabetic rats, we overexpressed DJ-1 C106S mutant protein in myocardial tissue of diabetic rats by AAV9-CMV-C106S DJ-1 injection. The results showed that IPostC did not improve cardiac function in diabetic rats with the overexpression of DJ-1 C106S mutant protein as compared with overexpressing DJ-1 protein, nor did it reduce infarct size **(**Fig. 2D**)**, CK-MB (Supplement 2A) and apoptosis level **(**Fig. 2G**)**. Furthermore, combination of DJ-1 overexpression and IPostC significantly reduced cardiac and serum 15-F2t-isoprostane levels **(**Fig. 2E-F**)**, superoxide anion production (Supplement 4B) and lipid hydroperoxide (Supplement 4D), but these effects of DJ-1 overexpression were not seen in the group in rats treated with DJ-1 C106S mutant. This indicates that the role of DJ-1 in restoring IPostC protection in diabetic rats needs its functional C106 site.

We next investigated the effect of IPostC on DJ-1 subcellular location. DJ-1 overexpression alone did not significantly affect phosphorylation of Akt and STAT3 **(**Fig. 2B**)**, nuclear Nrf2 and HO-1 **(**Fig. 2C**)** expression nor did it increase DJ-1 in the mitochondrial and nuclear fractions despite that the whole cell DJ-1 expression was increased **(**Fig. 2A**)**. However, DJ-1 overexpression in combination with IPostC significantly increased the phosphorylation level of Akt and STAT3, Nrf2/HO-1 pathway activity and promoted the translocation of DJ-1 to both nucleus and mitochondria. By contrast DJ-1 C106S mutant failed to preserve IPostC cardiac protection and had no significant effect on Nrf2/HO-1 and *p*-Akt/Akt ratio, *p*-STAT3/STAT3 ratio **(**Fig. 2B-C**)**. The data suggested that DJ-1 restoration of IPostC mediated myocardial protection in diabetic rats needed its functional C106 site.

### PTEN and Nrf2 regulated signals is responsible for aggravated MI/R injury in DJ-1 knockdown diabetic hearts

**Fig. 3:**
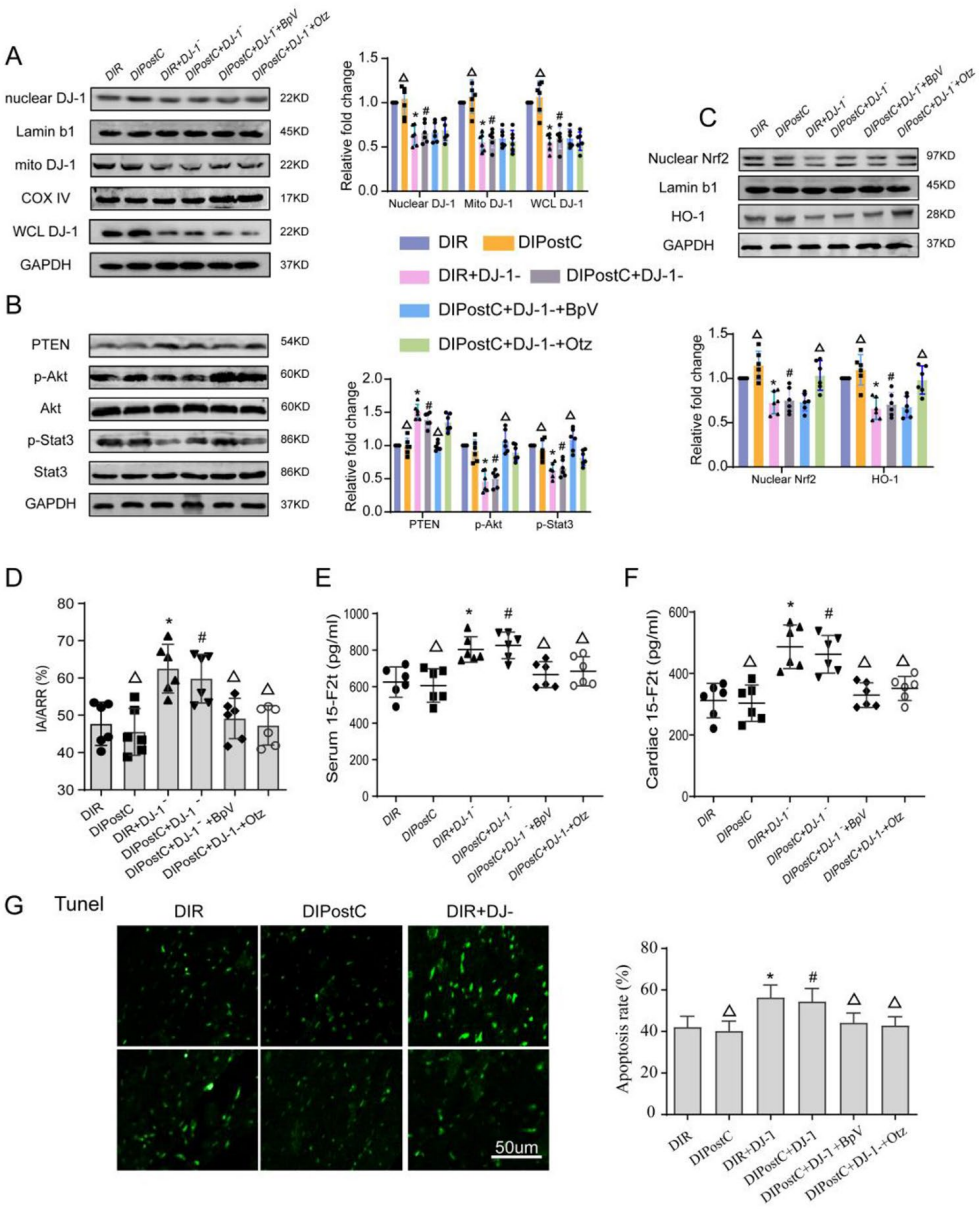
PTEN and Nrf2 regulated signals is responsible for aggravated MI/R injury in DJ-1 knockdown diabetic hearts (**A**) Representative Western blot images of mitochondrial, nuclear and whole cell DJ-1 expressions in the myocardium of the 6 groups of rats. (**B**) Representative Western blot images of PTEN, p-AKT and p-STAT3 expressions in the myocardium of the 6 groups of rats. (**C**) Representative Western blot images of nuclear Nrf2 and HO-1 expressions in the myocardium of the 6 groups of rats. (**D**) The myocardial infarction area was detected by TTC. (**E**–**F**) The serum and cardiac levels of 15-F2t-isoprostane was detected. (**G**) The tunnel staining for cell apoptosis rates were detected in different rat groups. Values are expressed as mean ± SD (*n* = 6 per group). *P < 0.05 vs. DIR group, ^#^*P* < 0.05 vs. $\text{DIPostC}\,\text{group,}\,^{\blacktriangle}$ *P* < 0.05 vs. DIPostC + DJ-1^−^ group

We subsequently investigated the effect of the DJ-1 knockdown and IPostC on MI/R injury in diabetic rats, we knocked down DJ-1 in myocardial tissue of diabetic rats by AAV9-DJ-1 siRNA injection. As shown in the (Supplement 3B), 3 weeks after AAV9-DJ-1 siRNA infection, the expression level of DJ-1 expression was decreased by 41% compared with control group. As shown in Table 2, in diabetic rats, HR, LVSP, +dp/dt and -dp/dt were further decreased after MI/R injury in the presence of DJ-1 gene knockdown, as demonstrated by increases in infarct size **(**Fig. 3D**)**, serum CK-MB (Supplement 3A) and apoptosis rate **(**Fig. 3G**)**.

To further explore the underlying mechanism of DJ-1 mediated cardioprotection, PTEN inhibitor BpV and Nrf2 agonist Otz were used in the ensuing study. IPostC combined with BpV or Otz, significantly attenuated MI/R injury in DJ-1 knockdown diabetic rats as demonstrated by reductions in infarct size **(**Fig. 3D**)**, serum CK-MB (Supplement 3A) and apoptosis rate **(**Fig. 3G**)** and cardiac and serum 15-F2t-isoprostane levels **(**Fig. 3E-F**)**, superoxide anion production (Supplement 5B) and lipid hydroperoxide (Supplement 5D) than DJ-1 knockdown group.

In DJ-1 knockout group, the expression levels of PTEN was increased **(**Fig. 3B**)**, while p-Akt/Akt, p-STAT3/STAT3 ratio **(**Fig. 3B**)**, nuclear Nrf2 and HO-1 expression **(**Fig. 3A**)** were decreased. Meanwhile, BpV administration reversed DJ-1 knockdown mediated reductions in PTEN and p-STAT3 expression **(**Fig. 3B**)**, but BpV administration had no significant effect on Nrf2 and HO-1 expression **(**Fig. 3C**)**. By contrast, Otz administration reversed DJ-1 knockdown mediated reductions in the expression of Nrf2 and HO-1 (all *p* < 0.05, DIPostC + DJ-1-+BpV or Otz vs. DIPostC + DJ-1-), but there were no significant change in the expression of PTEN and p-Akt/Akt, p-STAT3/STAT3 ratio. It is worth noting that the expression of p-Akt was decreased whenever IPostC was applied in combination with either BpV or Otz **(**Fig. 3B**)**. These data suggested that the aggravation of the MI/R injury in diabetic rats caused by DJ-1 knockdown could be partially or completely reversed by IPostC when applied in combination with either BpV or Otz, indicating that DJ-1 may be an upstream target of PTEN and Nrf2 regulated signals.

### H9c2 cells cultured in HG conditions are vulnerable to H/R injury but not responsive to HPostC

**Fig. 4:**
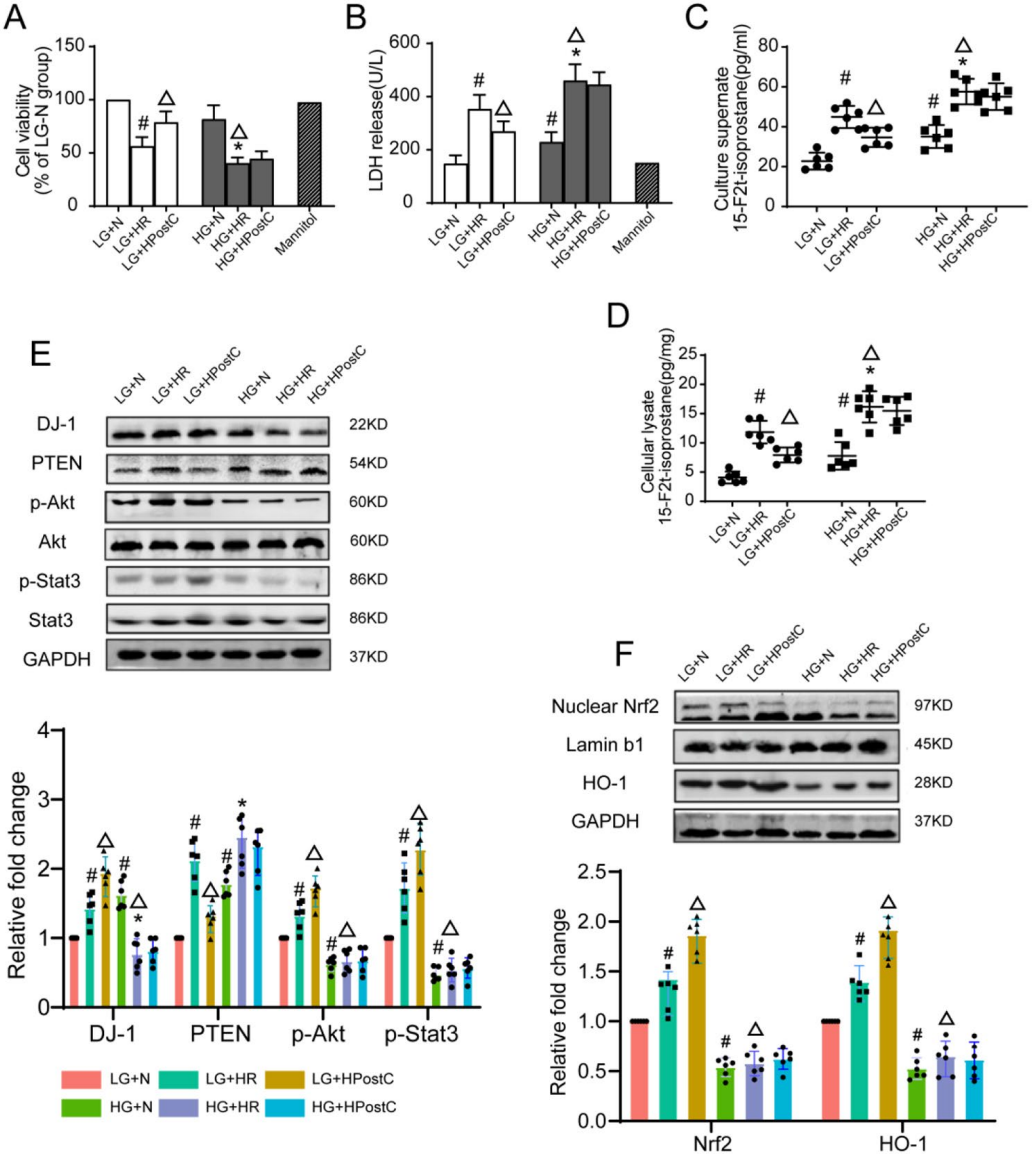
H9c2 cells cultured in HG conditions are vulnerable to H/R injury but are not responsive to HPostC (**A**) Cell viability was detected by CCK8. (**B**) The level of LDH was detected. (**C**–**D**) Culture supernate of 15-F2t-isoprostane and cell lysate level of 15-F2t-isoprostane. (**E**) Representative Western blot images of DJ-1, PTEN, p-AKE and p-STAT3 expressions in the myocardium of the 6 groups of rats. (**F**) Representative Western blot images of nuclear Nrf2 and HO-1 expressions in the myocardium of the 6 groups of rats. Values are expressed as mean ± SD (*n* = 6 per group). ^#^*P* < 0.05 vs. LG + $\text{N}\,\text{group,}\,^{\blacktriangle}$ *P* < 0.01 vs. LG + HR group, ^*^P < 0.05 vs. HG + N group

Additional investigations were performed in embryonic rat cardiomyocyte-derived H9c2 cells. Compared to the LG + N group, high glucose stimulation significantly reduced cell viability **(**Fig. 4A**)**, increased LDH release **(**Fig. 4B**)** and the levels of 15-F2t-isoprostane in the culture medium and in cardiomyocytes **(**Fig. 4C, D**)**. These effects were further exacerbated when cells were subjected to hypoxia-reoxygenation (H/R). HPostC significantly reversed these effects under LG conditions but failed to do so under HG conditions. Moreover, administration of the osmotic control mannitol did not affect cell viability and oxidative stress levels in this experiment.

As shown in **(**Fig. 4E**)**, DJ-1 expression levels in the HG + N group were significantly higher than in LG + N group, this increasement may have been a compensatory response of H9c2 cells to HG exposure. However, DJ-1 expression was dramatically decreased after H/R. By contrast, HPostC significantly increased DJ-1 expression in the LG condition, but not in the HG condition. Furthermore, H/R injury increased PTEN **(**Fig. 4E**)**, nuclear Nrf2 and HO-1 expression **(**Fig. 4F**)** and activated the phosphorylated Akt and STAT3 **(**Fig. 4E**)** in LG + N group, which were increased further by HPostC, however these alternations were not observed in H9c2 cells cultured under high glucose.

### DJ-1 overexpression preserves the protective effects of HPostC in HG-cultured H9c2 cells, whereas DJ-1 C106S mutant overexpression does not

**Fig. 5:**
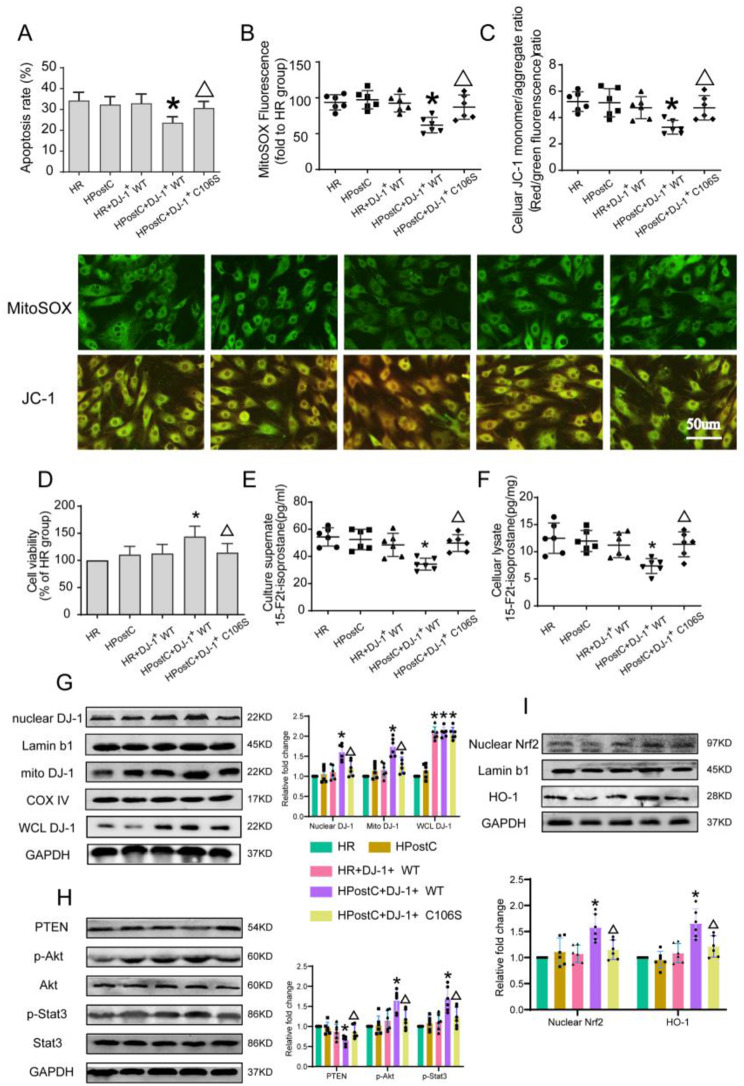
DJ-1 overexpression preserves the protective effects of HPostC in HG-cultured H9c2 cells, whereas DJ-1 C106S mutant overexpression does not (**A**–**C**) Apoptosis rates, MitoSOX fluorescence, Celluar JC-1 monemer/aggregate ratio in H9c2 cardiomyocytes pretreated with pcDNA3.1-DJ-1-Flag after 4 h hypoxia followed by 2 h reoxygenation with or without HPostC under 48 h HG stimulation, in the presence or absence of treatments. (**D**) Cell viability was detected by CCK8. (**E**–**F**) Culture supernate of 15-F2t-isoprostane and cell lysate level of 15-F2t-isoprostane. (**G**) Representative Western blot images of mitochondrial, nuclear and whole cell DJ-1 expressions of the 5 groups in H9c2 cells. (**H**) Representative Western blot images of PTEN, p-AKT and p-STAT3 expressions of the 6 groups in H9c2 cells. (**I**) Representative Western blot images of nuclear Nrf2 and HO-1 expressions in H9c2 cells. Values are expressed as mean ± SD (*n* = 6 per group). *P < 0.05 vs. $\text{HR}\,\text{group;}\,^{\blacktriangle}$ *P* < 0.05 vs. HR + DJ-1^+^ WT group

To determine whether DJ-1 preserves the cardioprotective effect of HPostC in H9c2 cells exposed to HG, we overexpressed DJ-1 by transfected pcDNA3.1-DJ-1-Flag plasmid in H9c2 cells. Biochemically, the expression level of DJ-1 in the pcDNA3.1-DJ-1-Flag transfected group was nearly 3.8 times higher than that in the vector group (Supplement 4A). DJ-1 overexpressed combined with HPostC significantly attenuated H/R injury as demonstrated by decreased apoptosis rate **(**Fig. 5A**)** and increased cell viability **(**Fig. 5D**)**. Moreover, DJ-1 overexpression combined with HPostC significantly attenuated the extent of oxidative stress in H9c2 cells exposed to HG evidenced by decreased O_2_^−^ (Supplement 4B), H_2_O_2_ (Supplement 4C) as well as cell and medium 15-F2t-isoprostane levels **(**Fig. 5E-F**).** In addition, we estimated the effect of DJ-1 overexpression on mitochondrial oxidative stress. MitoSOX fluorescence intense and JC-1 monomer/aggregate ratio was decreased in cells with DJ-1 overexpression and concomitantly HPostC treated group **(**Fig. 5B-C**)**. All the beneficial effect of DJ-1 overexpression in HPostC group was abrogated in the DJ-1 mutant (C106S) transfected group which indicates that C106 site of DJ-1 is crucial for DJ-1 mediated HPostC protection.

In the meantime, we investigated the effect of HPostC and DJ-1 overexpression on the levels of nuclear and mitochondrial DJ-1, PTEN, Nrf2, HO-1 and phosphorylated Akt and STAT3 levels in H9c2 cells cultured under high glucose conditions. DJ-1 overexpression alone did not significantly affect phosphorylation of Akt and STAT3 **(**Fig. 5H**)**, nuclear Nrf2 and HO-1 **(**Fig. 5I**)** expression nor DJ-1expression in mitochondrial and nuclear fraction **(**Fig. 5G**)** when H9c2 cells were subjected to H/R injury under HG exposure. However, DJ-1 overexpression in combination with HPostC significantly increased the phosphorylation level of Akt and STAT3, nuclear Nrf2 and HO-1 expression and promoted the translocation of DJ-1 to both nucleus and mitochondria. In the DJ-1 mutant study, DJ-1 C106S mutant failed to preserve HPostC cellular protection and had no significant effect on Nrf2/HO-1 and RISK pathway activity. The data suggested that the protective effect of DJ-1 on the recovery of HPostC in H9c2 cells exposed to HG might be related to its C106 Site.

### DJ-1 knockdown aggravated HR injury in HG-cultured H9c2 cells which was partially reversed by PTEN inhibition or Nrf-2 activation

**Fig. 6:**
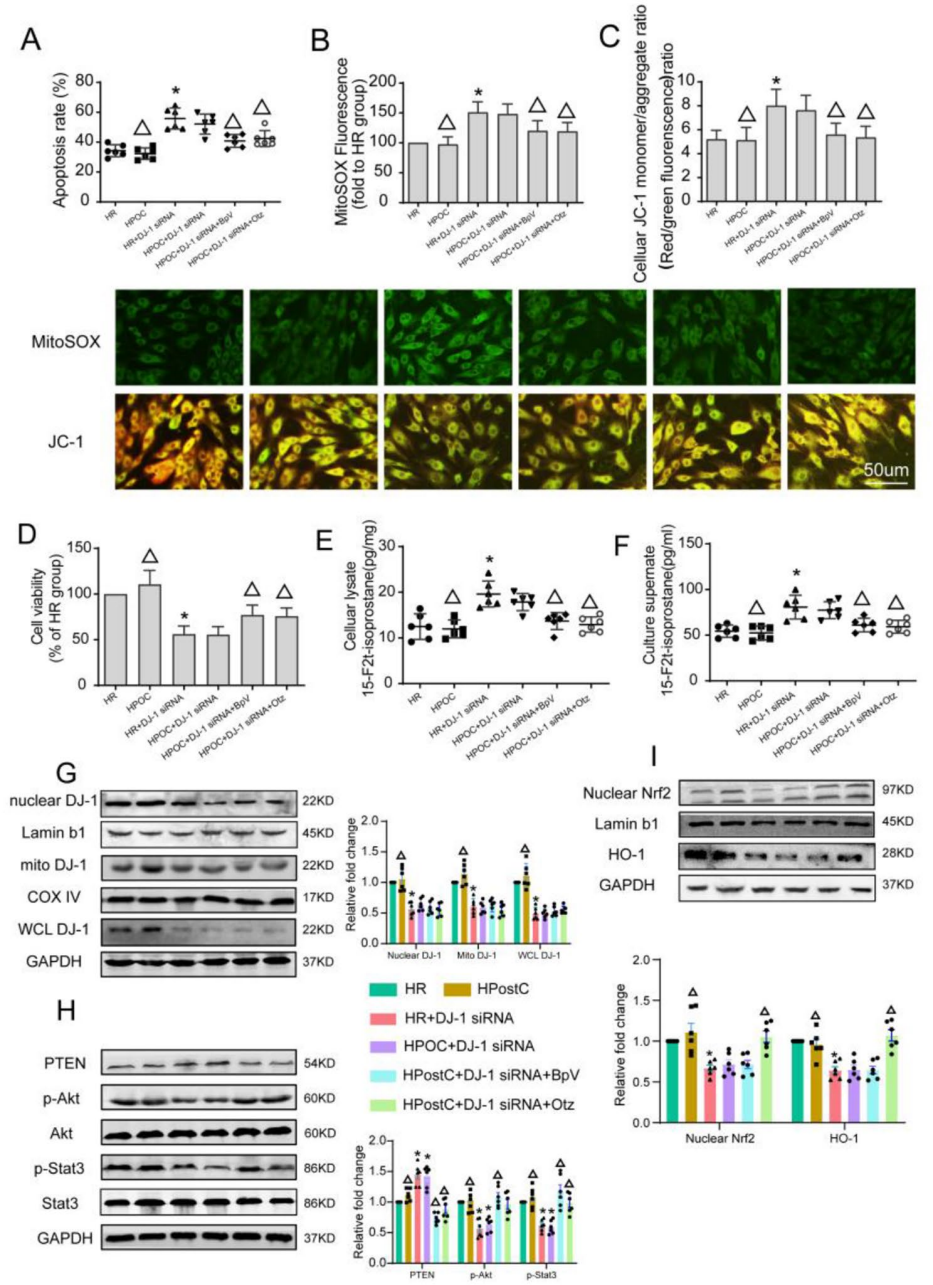
DJ-1 knockdown aggravated HR injury in in HG-cultured H9c2 cells which was partially reversed by PTEN inhibition or Nrf-2 activation (**A**–**C**) Apoptosis rates, MitoSOX fluorescence, Celluar JC-1 monemer/aggregate ratio in H9c2 cardiomyocytes pretreated with pcDNA3.1-DJ-1-Flag after 4 h hypoxia followed by 2 h reoxygenation with or without HPostC under 48 h HG stimulation, in the presence or absence of treatments. (**D**) Cell viability was detected by CCK8. (**E**–**F**) Culture supernate of 15-F2t-isoprostane and cell lysate level of 15-F2t-isoprostane. (**G**) Representative Western blot images of mitochondrial, nuclear and whole cell DJ-1 expressions of the 6 groups in H9c2 cells. (**H**) Representative Western blot images of PTEN, p-AKT and p-STAT3 expressions of the 6 groups in H9c2 cells. (**I**) Representative Western blot images of nuclear Nrf2 and HO-1 expressions in H9c2 cells. Values are expressed as mean ± SD (*n* = 6 per group). *P < 0.05 vs. $\text{HR\,group;}\,^{\blacktriangle}$ *P* < 0.05 vs. HR + DJ-1 siRNA group

To further confirm whether downregulation of DJ-1 contributes to the loss of HPostC in H9c2 cells exposed to HG, we transfected H9c2 cells with DJ-1 siRNA which reduced the DJ-1 protein expression level by 68% as compared with the control group (Supplement 5A). DJ-1 siRNA transfection aggravated the damage caused by H/R in H9c2 cells exposed to HG as demonstrated by decreased cell viability **(**Fig. 6D**)**. Moreover, HPostC did not confer protection against H/R injury in DJ-1 knockdown H9c2 cells. Then we used the PTEN inhibitor BpV and Nrf2 agonist Otz to further explore the signaling pathway of DJ-1 cardioprotection. HPostC combined with BpV or Otz, significantly attenuated H/R injury in DJ-1 knockdown H9c2 cells despite the knockdown of DJ-1 with DJ-1 siRNA as demonstrated by increased cell viability **(**Fig. 6D**)**, decreased apoptosis rate **(**Fig. 6A**)**. Moreover, HPostC combined with BpV or Otz, significantly attenuated the extent of oxidative stress in DJ-1 knockdown H9c2 cells exposed to HG as evidenced by decreased O_2_^−^, and H_2_O_2_(Supplement 5B-C) as well as cell and medium 15-F2t-isoprostane levels **(**Fig. 6E-F**)**. In addition, we estimated the effect of DJ-1 knockdown and HPostC on mitochondrial oxidative stress. MitoSOX fluorescence intense and JC-1 monomer/aggregate ratio was decreased in HPostC combined BpV or Otz group **(**Fig. 6B-C**)**.

We next investigated the effect of HPostC and DJ-1 knockdown on expression levels of nuclear and mitochondrial DJ-1, PTEN, Nrf2, HO-1 and phosphorylated Akt and STAT3 level in H9c2 cells cultured under high glucose conditions. In DJ-1 knockdown group, the expression levels of PTEN **(**Fig. 6H**)** was increased, while nuclear Nrf2 and HO-1 **(**Fig. 6I**)** expression, p-Akt/Akt and p-STAT3/STAT3 **(**Fig. 6H**)** ratio levels decreased in H9c2 cells cultured under high glucose conditions. Meanwhile, BpV in combination with HPostC reduced PTEN expression and increased p-Akt/Akt and p-STAT3/STAT3 ratio, but the expression of Nrf2 and HO-1 did not change significantly as compared with the group of HR plus DJ-1 siRNA gene knockdown. However, in the Otz and HPostC jointly treated group, the expression of Nrf2 and HO-1 were increased **(**Fig. 6H**)**, but there were no significant change in the expression of PTEN compare to HPostC + DJ-1 siRNA group. These data suggested that the aggravation of the H/R injury in H9c2 cells exposed to HG caused by DJ-1 knockdown could be partially or completely reversed by HPostC when applied in combination with either BpV or Otz, indicating that DJ-1 may be an upstream target of PTEN and Nrf2 regulated signals.

## Discussion

In the present study, we demonstrated that reduction of cardiac DJ-1 in diabetic hearts resulted in reduced sensitivity of the myocardium to IPostC-induced protection and that DJ-1 overexpression restored the cardioprotective effects of IPostC in diabetic rats. This protective effect of DJ-1 overexpression in combination with IPostC was accompanied by PTEN inactivation and translocation of DJ-1 from the cytoplasm to the nucleus and mitochondria followed by further increases of Nrf-2/HO-1 transcription and mitochondrial activity. These beneficial effects were not observed in rats transfected with the DJ-1 C106S mutant. To the best of our knowledge, this study is the first to show that DJ-1 restores the protective effect of IPostC by inhibiting PTEN expression and promoting DJ-1 translocation to the mitochondria and nucleus in the diabetic myocardium.

Previous study showed that IPostC in combination with cyclosporine-A but not IPostC alone applied at the onset of reperfusion reduces I/R injury in the diabetic heart, and reduced levels of oxidative stress are associated with significant cardioprotection [22], but the underlying did not explored. The DJ-1 protein is believed to have several functions, but it has been consistently found to antagonize oxidative stress. DJ-1 contains three cysteine residues at positions 46, 53 and 106 [23]. Of the three cystein residues, C106 is highly susceptible to oxidative stress, and the oxidative status of C106 determines the activity level of DJ-1, suggesting that C106 is essential for the complete function of DJ-1 [24]. In the present study, we found that DJ-1 protein expression was decreased in diabetic rats at baseline. In non-diabetic rats, IPostC significantly improved cardiac function, attenuated MI/R injury, significantly increased DJ-1 expression and decreased ROS production, but the protective effects of IPostC were abolished in the hearts of diabetic rats. Meanwhile, AAV9-mediated cardiac DJ-1 overexpression restored the sensitivity of diabetic hearts to IPostC-induced cardioprotection. Furthermore, we also found that the DJ-1 C106S mutant construct abolished the cytoprotective activity of IPostC against I/R injury in diabetic rats. These results suggest that decreased DJ-1 expression may be responsible for the diminished cardioprotective effects of IPostC in diabetes and that the C106 site of DJ-1 plays a crucial role in DJ-1-mediated cardiac protection.

PTEN was originally identified as a tumor suppressor gene frequently lost from a region of chromosome 10q23 in a variety of human tumors [25], and two major mechanisms underlying the posttranslational regulation of PTEN are phosphorylation and oxidation [26]. Our previous study revealed that PTEN inhibition restores IPostC-induced cardioprotection in diabetes, mainly through upregulating the PI3K/Akt signaling pathway and partially by upregulating the JAK2/STAT3 signaling pathway, ultimately activating the GSK-3β-mediated mitochondrial pathway; this suggests that PTEN is a target for restoring the effects of IPostC [27]. DJ-1 has been shown to negatively regulate PTEN and activate the PI3K/Akt pathway [28]. Our study revealed that DJ-1 overexpression inhibits PTEN expression and activates the PI3K/Akt pathway, restoring the cardioprotective effects of IPostC. However, mutations at the DJ-1 C106 site of DJ-1 lack this effect. The underlying mechanism may be related to the oxidative status of DJ-1, the C106 residue determines the activity level of DJ-1. Cumulative experimental and clinical studies have shown that diabetic patients have increased vulnerability to MI/R injury and that lipid peroxidation mediated by ROS during I/R is key to the connection between diabetes and MI/R injury. In the present study, we demonstrated that AAV-9-mediated cardiac DJ-1 inhibition aggravated the effects of I/R injury in diabetic hearts and that the combination of AAV-9 DJ-1 siRNA and IPostC did not confer cardioprotection in diabetic hearts. In the in vitro study, DJ-1 overexpression, but not DJ-1 knockdown, restored HPostC-induced protection in H9c2 cells subjected to HR injury under HG conditions. Moreover, PTEN inhibition induced by BpV did not confer significant protection in DJ-1 knockdown H9c2 cells. Thus, we conclude that PTEN inhibition is essential for the restoration of the DJ-1 mediated cardioprotective effects of IPostC in the diabetic heart.

DJ-1 is mainly localized in the cytosol and translocates to the mitochondria and nucleus to protect against oxidative stress-induced cell death under oxidative conditions [29]. In the present study, we demonstrated that IPostC significantly induced DJ-1 mitochondrial translocation in nondiabetic rats but that DJ-1 mitochondrial translocation was blocked in diabetic hearts during I/R injury. DJ-1 mitochondrial translocation was restored in DJ-1-overexpressing diabetic rats. Further study showed that the DJ-1 C106S mutant abolished the cytoprotective effect of IPostC against I/R injury in diabetic rats. However, the C106S mutant did not affect DJ-1 mitochondrial translocation.

Moreover, DJ-1 also functions as an antioxidant by upregulating the expression of other antioxidant genes, such as the transcriptional factor Nrf-2. In the current study, we found that IPostC caused significant DJ-1 nuclear translocation and increased nuclear Nrf-2 expression. However, nuclear translocation during I/R injury was blocked in diabetic hearts. Further study showed that DJ-1 nuclear translocation was restored in DJ-1-overexpressing rats. DJ-1 C106S mutant overexpression abolished the cytoprotective effect of IPostC against I/R injury in diabetic rats. However, the C106S mutant did not affect DJ-1 nuclear translocation. These results suggest that the C106 residue of DJ-1 is essential for DJ-1-mediated cardioprotection.

## Conclusions

We demonstrate that in diabetes, DJ-1 inhibition contributes to abolishing the cardioprotective effects of IPostC in STZ-induced type 1 diabetic rats. DJ-1 overexpression restores IPostC-induced cardioprotection in diabetes, mainly through inhibiting PTEN and translocating to the mitochondria and nucleus in the diabetic myocardium. Therefore, DJ-1 overexpression may be an effective therapy for restoring myocardial responsiveness to IPostC in diabetes.

### Electronic supplementary material

Below is the link to the electronic supplementary material.

Supplementary Material 1

## Data Availability

The datasets used and analysed during the current study are available from the corresponding author on reasonable request.
