# Supplementary material for: DJ-1 preserves ischemic postconditioning-induced cardioprotection in STZ-induced type 1 diabetic rats: role of PTEN and DJ-1 subcellular translocation
Source: Cell Commun Signal. 2024 May 2;22:252. doi: 10.1186/s12964-024-01638-2 (PMC11064239; doi:10.1186/s12964-024-01638-2)
Supplement: Supplementary file 1 — Supplementary Material 1 [file 12964_2024_1638_MOESM1_ESM.docx]

Supplement 1


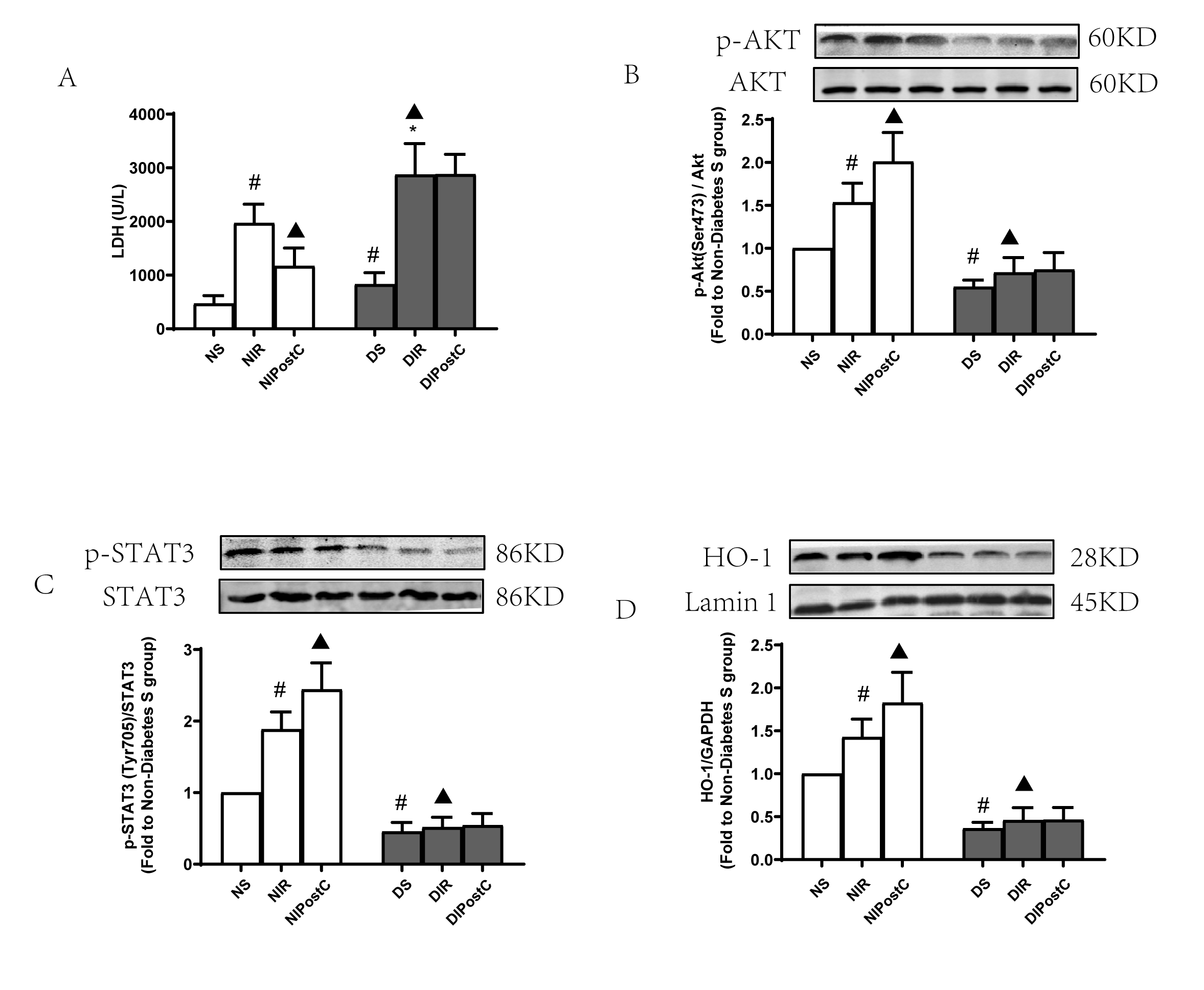


**Supplement 1** (A) Serum levels of LDH. (B-D) Representative Western blot images of p-AKT, p-STAT3 and HO-1 expressions in the myocardium of the 6 groups of rats. Values are expressed as mean ± SD (n= 6 per group). ^#^*P*<0.05 vs. NS group, ^▲^*P*<0.05 vs. NIR group, ^*^*P*<0.05 vs. DS group.

**Supplement 2**


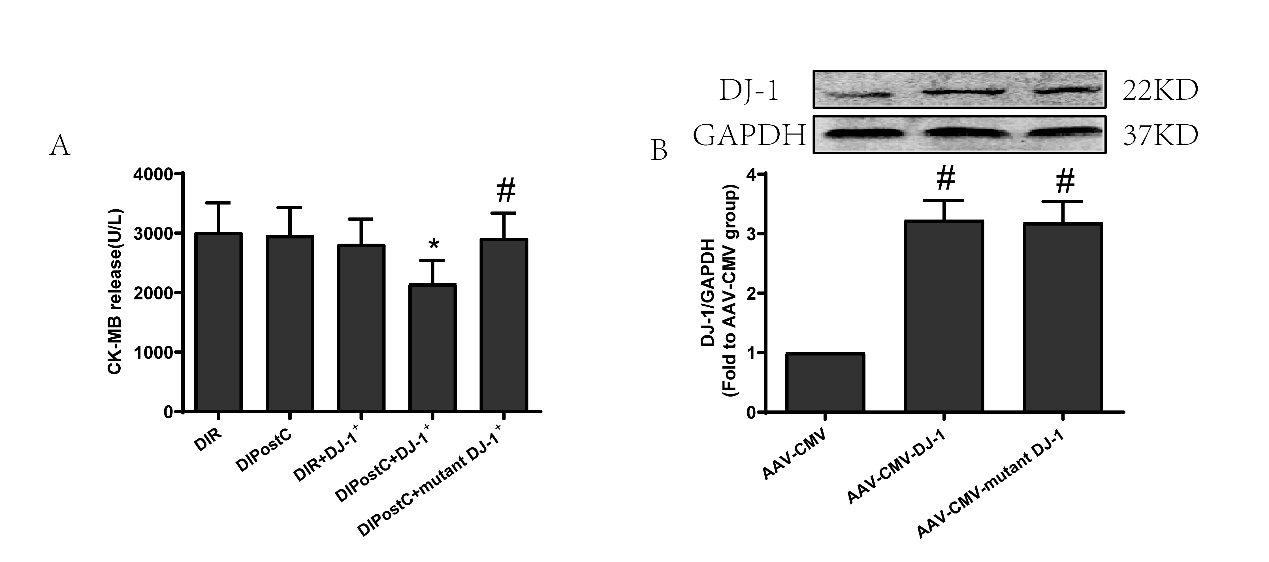


**Supplement 2** (A) The CK-MB in serum was detected. Values are expressed as mean ± SD (n= 6 per group). **P*<0.05 vs. DIR group, **^#^***P*<0.05 vs. DIPostC+DJ-1^+^ group. (B) DJ-1 protein expression in the hearts after AVV-AMV-DJ-1 injection. Values are expressed as mean ± SD (n= 6 per group). ^#^ *P*<0.05 vs. AAV-CMV group.

**Supplement 3**


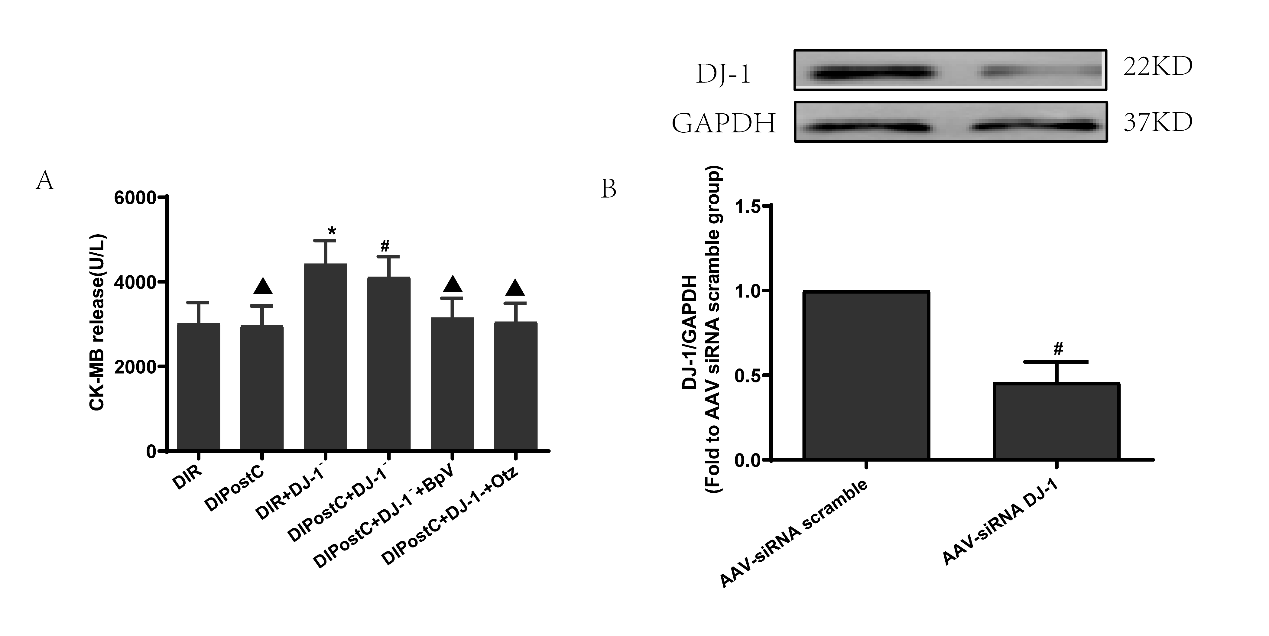


**Supplement 3** (A) The CK-MB in serum was detected. Values are expressed as mean ± SD (n= 6 per group). **P*<0.05 vs. DIR group, ^#^*P*<0.05 vs. DIPostC group, ^▲^*P*<0.05 vs. DIPostC+DJ-1^-^ group. (B) DJ-1 protein expression in the heart after AAV-siRNA DJ-1 injection. Values are expressed as mean ± SD (n= 6 per group). ^#^ *P*<0.05 vs. AAV-siRNA scramble group.

**Supplement 4**

**
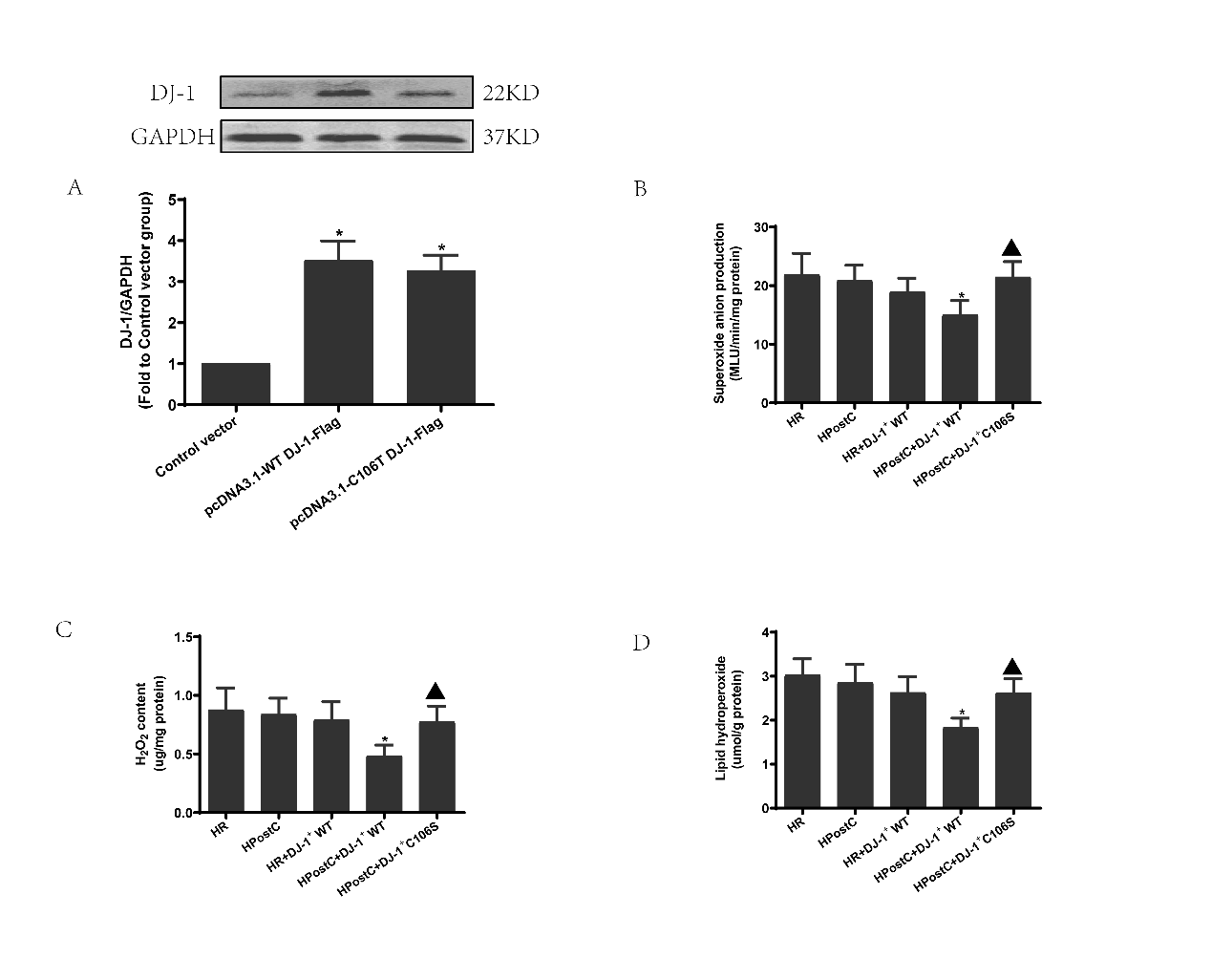
Supplement 4**  (A) DJ-1 protein expression in the H9c2 cells after pcDNA3.1-WT DJ-1 injection. Values are expressed as mean ± SD (n= 6 per group). ^*^ *P*<0.05 vs. siRNA scramble group. (B) Superoxide anion production. (C) H_2_O_2_ content. (D) Lipid Hydroperoxide. Values are expressed as mean ± SD (n= 6 per group). **P*<0.05 vs. HR group; ^▲^*P*<0.05 vs. HR+DJ-1^+^ WT group.

**Supplement 5**


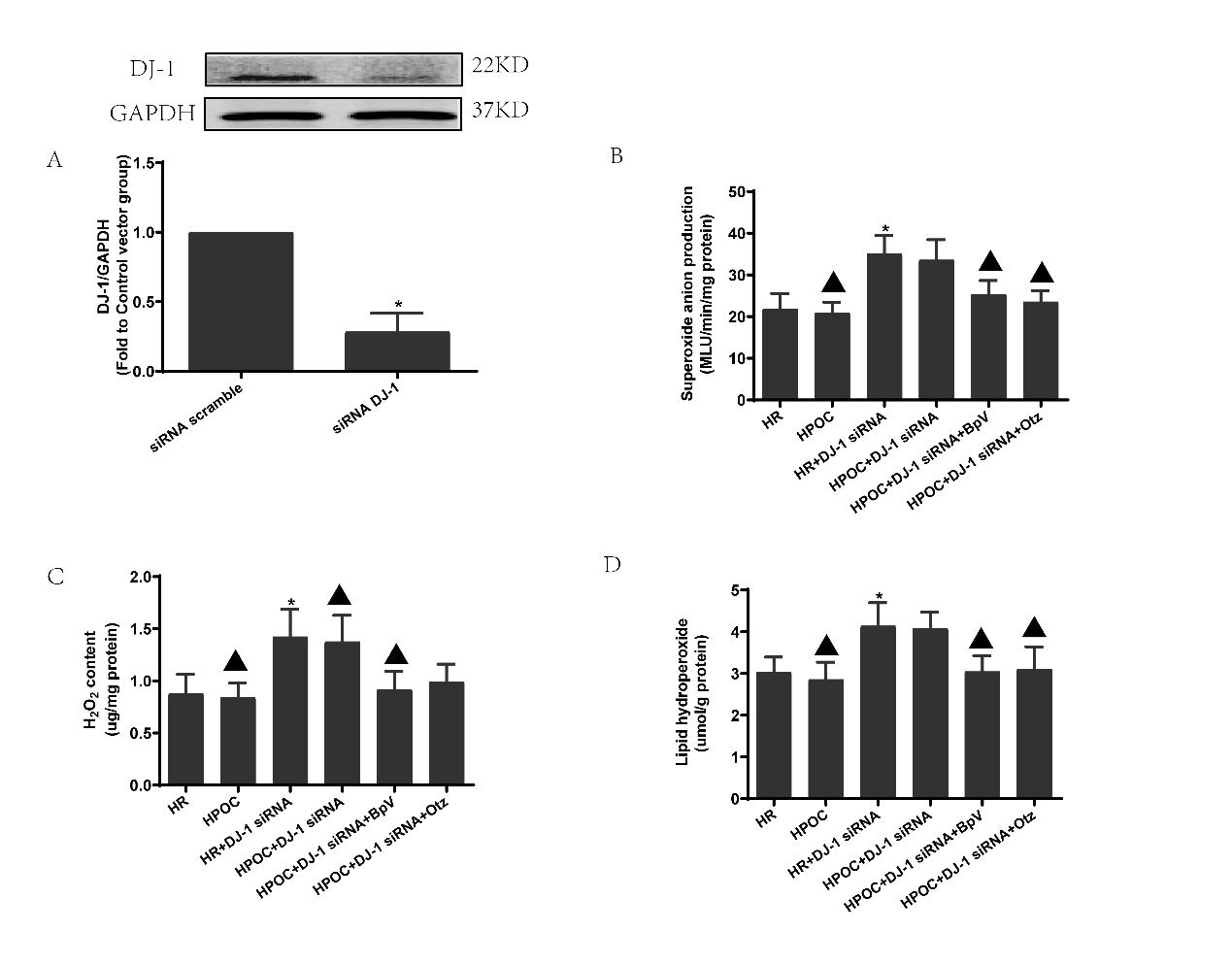


**Supplement 5** (A) DJ-1 protein expression in the H9c2 cells after siRNA DJ-1 injection. Values are expressed as mean ± SD (n= 6 per group). ^*^ *P*<0.05 vs. siRNA scramble group. (B) Superoxide anion production. (C) H_2_O_2_ content. (D) Lipid Hydroperoxide. Values are expressed as mean ± SD (n= 6 per group). **P*<0.05 vs. HR group; ^▲^*P*<0.05 vs. HR+DJ-1 siRNA group.
